# Ballistic femoral neck fractures: Current concepts in evaluation and management

**DOI:** 10.1051/sicotj/2026084

**Published:** 2026-10-23

**Authors:** Mohamed I. Abulsoud, Muhammad Atif, Mohamed H. Abdou, Mahmoud M. Hassaan, Zenat Khired

**Affiliations:** 1 Department of Orthopedic Surgery, Faculty of Medicine, Al-Azhar University Cairo Egypt; 2 Department of Orthopedic Surgery, King Khalid Hospital Najran Saudi Arabia; 3 Orthopaedic Division, Department of Surgery, College of Medicine, Jazan University Jazan Saudi Arabia

**Keywords:** Open fracture neck of femur, Ballistic femoral neck fractures, Total hip arthroplasty, Sliding hip screws, DHS, Cannulated screws

## Abstract

Ballistic femoral neck fractures are complex, high-energy injuries that require a management approach distinct from conventional blunt trauma. These current concepts review examines the evolving landscape of evaluation and treatment for these rare injuries based on contemporary literature. Initial management emphasizes the Advanced Trauma Life Support (ATLS) protocol, with a critical focus on identifying associated neurovascular and skeletal injuries, which are present in approximately one-third of cases. Diagnostic evaluation relies on plain radiography and thin-slice CT to delineate fracture morphology and bullet trajectory, with CT angiography recommended if vascular compromise is suspected. Surgical decision-making balances the goals of head preservation through open reduction and internal fixation (ORIF) against the definitive stability provided by total hip arthroplasty (THA). While ORIF remains the standard for young, active patients, clinicians must account for the historically high incidence of nonunion and infection associated with ballistic mechanisms. In cases of failed osteosynthesis or catastrophic articular comminution, conversion to THA offers a reliable salvage option with favorable functional outcomes. This review highlights the need for individualized treatment plans and meticulous clinical follow-up to manage the inherent risks of this challenging injury pattern.

## Introduction

Ballistic femoral neck fractures are rare but exceptionally challenging injuries typically resulting from high-energy gunshot trauma. Although uncommon, their clinical significance is profound; they frequently present in the context of polytrauma and are often complicated by concomitant vascular injuries, pelvic or acetabular fractures, gross soft-tissue contamination, and retained intra-articular fragments. Managing these injuries remains controversial, as surgeons must carefully balance the competing goals of achieving fracture stability, preserving the native hip, and mitigating the substantial risks of infection, avascular necrosis, and non-union [1].

Unlike blunt-trauma femoral neck fractures, ballistic injuries introduce the complex biology of an open fracture, including cavitary tissue damage and highly unpredictable fracture morphology. Consequently, treatment algorithms vary widely. Current strategies range from non-operative management for highly selected incomplete fractures to open reduction and internal fixation (ORIF) and to primary or conversion total hip arthroplasty for severe comminution or failed osteosynthesis. However, guiding evidence remains sparse, largely limited to small retrospective cohorts, mixed proximal femur series, and principles extrapolated from the broader blunt trauma literature [2, 3].

Given this clinical uncertainty, a dedicated synthesis of the current evidence is essential to clarify the evaluation protocols, injury patterns, treatment options, and complication profiles associated with ballistic femoral neck fractures. By summarizing contemporary literature, this review highlights practical considerations for initial assessment, operative decision-making, and expected outcomes to help guide the management of this uncommon but highly consequential injury.

## Methods of literature review

A focused literature search was conducted to identify published evidence regarding ballistic femoral neck fractures and related ballistic injuries of the proximal femur and hip. PubMed/MEDLINE, Scopus, and Google Scholar were searched using combinations of the following terms: “ballistic femoral neck fracture,” “gunshot femoral neck fracture,” “gunshot hip,” “ballistic proximal femur fracture,” “gunshot proximal femur fracture,” “femoral neck fracture,” “gunshot wound,” “open fracture,” “internal fixation,” and “total hip arthroplasty.” The search focused primarily on studies published in the contemporary literature. At the same time, earlier publications were included when they provided relevant foundational evidence regarding complications, debridement, retained intra-articular bullets, or arthroplasty. Reference lists of relevant articles were also screened to identify additional eligible publications.

Given the rarity of ballistic femoral neck fractures and the limited number of dedicated clinical studies, evidence from studies specifically addressing ballistic femoral neck fractures was prioritized. Where direct evidence was unavailable, relevant data from ballistic proximal femoral or hip injuries and from the broader femoral neck fracture and arthroplasty literature were considered and explicitly identified as extrapolated evidence. The available literature was qualitatively synthesized according to injury characteristics, diagnostic evaluation, operative management, fixation strategies, arthroplasty, complications, and functional outcomes. This review was designed as a narrative/current-concepts review and was not intended to constitute a formal systematic review or meta-analysis.

## Incidence and epidemiology

Ballistic femoral neck fractures remain uncommon even in high-volume trauma centers, with recent series reporting only small cohorts followed over several years [1, 2]. In the only dedicated cohort study focusing specifically on ballistic femoral neck fractures, 17 patients were identified over the study period, indicating a relatively low absolute incidence [1]. Affected patients are predominantly young males, with a reported median age between 20 and 30 years, reflecting the strong epidemiologic association with firearm-related trauma in this demographic [3].

## Associated injuries

Ballistic femoral neck fractures frequently occur in the context of polytrauma and are commonly accompanied by other vascular, skeletal, and soft-tissue injuries. [4] Approximately one-third of patients sustain a concomitant vascular injury, most often involving the femoral or profunda femoris arteries, which underscores the need for routine vascular assessment and selective angiography or CT angiography. [1, 2, 5] Nearly half of patients experience at least one additional osseous injury, commonly involving the pelvis, acetabulum, or other portions of the femur or lower extremity. Injury Severity Scores in these cohorts are typically in the mid-single digits to low-teens, reflecting moderate overall trauma burden despite the focal nature of the femoral neck injury [5].

## Evaluation

Gunshot injuries are typically high-energy trauma requiring multidisciplinary evaluation to assess for vascular, neurological, visceral, and musculoskeletal injuries [4–6]. All patients should be initially evaluated according to the Advanced Trauma Life Support (ATLS) protocol to stabilize the patient, control hemorrhage, and exclude life-threatening injuries [7], orthopaedic assessment should specifically address the neurovascular status of the lower limb, including palpable pulses, capillary refill, and signs of compartment syndrome, because ballistic femoral neck fractures are associated with vascular injury in up to one-third of patients [1, 2, 5]. Plain radiographs of the pelvis and hip (anteroposterior pelvis and lateral hip views) are obtained first to confirm the fracture pattern, degree of displacement, comminution, and the localization of the bullet if still retained, as well as to screen for associated pelvic or acetabular fractures. Whenever plain films are equivocal or suggest an occult or minimally displaced fracture, cross-sectional imaging with thin-slice CT is indicated to delineate fracture lines, articular extension, and bullet or fragment trajectory. Furthermore, CT angiography should be strongly considered if there is any suspicion of vascular injury or hemodynamic instability [4, 8]. Because ballistic fractures are open injuries, prophylactic antibiotics should be initiated as rapidly as possible. However, Available ballistic-fracture literature does not provide strong evidence supporting routine prophylaxis beyond 48 hours in uncomplicated cases; antibiotic duration should be individualized according to contamination, associated injuries, debridement adequacy, and established infection [9, 10].

## Treatment options

Current management of ballistic femoral neck fractures follows principles derived from blunt-trauma femoral neck fractures. Still, it is adapted to the unique features of high-energy gunshot injuries (soft-tissue damage, comminution, and vascular compromise) [3, 4]. Three main strategies are described in the literature: non-operative management, open reduction and internal fixation (ORIF), and acute or early total hip arthroplasty (THA) [6].

## Non-operative treatment

Non-operative management is reserved for incomplete fractures in carefully selected patients, such as those with severe comorbidities, non-ambulatory status, or where surgical risk outweighs potential benefit. In the ballistic-fracture cohort reported by Harrison et al., three patients with incomplete fractures were managed non-operatively. However, long-term follow-up and formal outcome metrics for this subgroup are limited. [1] Given the high-energy nature of ballistic trauma and the risk of delayed displacement, non-operative treatment is typically reserved for highly specific cases. It does not represent the standard clinical approach. [6]

## Open reduction and internal fixation (ORIF)

Open reduction and internal fixation (ORIF) remains the preferred head-preserving strategy in young patients when anatomical/stable reconstruction is feasible. [1, 3]. In mixed cohorts of open proximal femoral fractures caused by gunshot wounds, which include a subset of femoral neck injuries, ORIF remains the predominant treatment strategy [5]. Parallel data from a recent comparison of civilian ballistic and blunt neck-of-femur fractures show broadly similar rates of non-union, infection, and reoperation between the two mechanisms [8]. These findings suggest that high-energy ballistic injuries should be managed with the same emphasis on stable fixation, soft-tissue preservation, and close follow-up as blunt trauma injuries [8].

When focusing specifically on fractures treated with ORIF, the available evidence highlights relatively modest healing success and a higher risk of non-union than traditionally expected for blunt-trauma femoral neck fractures in young adults. In a ballistic-specific cohort, roughly 60–62% of fractures managed with ORIF healed, while the remainder developed non-union or other complications [1]. A separate comparative cohort study of 12 ballistic and 16 blunt femoral neck fractures found similar overall non-union rates (about 25% in each group) [2]. Furthermore, contemporary multicenter retrospective data show that ballistic femoral neck fractures treated with operative repair have high failure rates, with 5–7 patients who met follow-up criteria failing due to non-union [11]. The authors concluded that ballistic femoral neck fractures remain associated with a substantial risk of non-union. They emphasized the need for larger multicenter studies to define the optimal treatment strategy [11].

From a technical standpoint, the removal of retained intracapsular bullets during ORIF is generally recommended. Lead arthropathy, driven by dissolved lead and its chondrotoxic effects, along with systemic lead poisoning, has been documented in several case reports, making meticulous joint debridement a critical step during initial fixation [12, 13].

**Synthesis:** Based on the available evidence, there is no clear superiority of one implant over another for ballistic femoral neck fractures, with cannulated screws and sliding hip screws (SHS) being the most frequently utilized [1, 2, 4, 5, 8, 11]. Healing, when it occurs, typically becomes evident within the first 12 months, with most failures manifesting within this same timeframe [6, 14]. However, clinicians must anticipate a high complication burden: Reported union rates vary substantially across small retrospective cohorts, ranging from approximately 29% to 75%, although direct comparison is limited by heterogeneity in injury patterns, treatment strategies, follow-up, and outcome definitions; nearly 40% of patients experience at least one postoperative complication, notably non-union or infection (Table 1).

**Table 1:** Summarizes the studies that included ballistic femoral fractures and their main findings.

Study (author, year)	(*N*)	AO subclass (B1​/B2​/B3​)	Garden subclass (I/II/III/IV)	Pauwels subclass (I/II/III)	Implants used for fixation (*n*)	Union Rate	Complications
Harrison et al., 2025 [1]	13	B1​:1, B2​:3, B3​:9	I:4, IV:9	II:3 III:10	SHS: 6 CS: 3 CMN: 2 Hybrid: 2	62%	38%
Serotte et al., 2024 [2]	12	31B (all)	I:4, II:3 III:3 IV:2	N/A (Mean 51°)	SHS: 6 FNS: 5 CS: 1	75%	25%
Maqungo et al., 2020 [4]	9	N/A	N/A	N/A	N/A	66%	33%
Hutaif et al., 2024 [5]	4	B1​:2, B2​:2	N/A	N/A	CS: 3, Plate: 1	75%	42%
Farris et al., 2025 [8]	6	B1​:1, B2​:2, B3​:3	N/A	N/A	CMN/Plates: 6	60%	28%
Shu et al., 2024 [11]	14	31B (all)	N/A	N/A	CS: 8 SHS: 6	29%	71%
Aggregate of reported cohorts	58	B1​:4 B2​:9 B3​:15	I:8, II:3, III:3, IV:12	II:3 III:10	CS: 18 (31%) SHS: 18 (31%) FNS: 5 (8.6%) CMN: 4 (6.9%) Hybrid: 2 (3.5%) Plates (medial or proximal femur: 2 (3.5%) Unspecified: 9 (15.5%)	58%	42%

Abbreviations: *N* = number of cases of balistic femoral neck of femur in the study, N/A = not available, SHS = sliding hip screw, Cs = cannulated screws, CMN = cephalomedullary nail, FNS = femoral neck system.

## Arthroplasty

### Ballistic-specific evidence

Evidence regarding arthroplasty following ballistic injuries to the hip remains limited but has expanded in recent years. Evidence from the broader non-ballistic literature indicates that THA performed for femoral neck fractures carries a higher complication burden than elective THA performed for osteoarthritis [15]; however, these findings cannot be directly extrapolated to ballistic injuries. The largest contemporary series specifically evaluating total hip arthroplasty (THA) following civilian gunshot injuries to the hip included 14 patients, all male, with a mean age of 32 years. Ten patients underwent acute primary THA, whereas four underwent delayed THA following failed open reduction and internal fixation (ORIF). The mean interval from injury to surgery was 7.6 days in the acute group and 39 months in the delayed group. One patient (7%) in the acute group developed an early prosthetic joint infection; this patient had an associated large-bowel injury, although there was no direct communication between the bowel injury and the hip joint. No prosthetic joint infection occurred in the delayed group. Among patients undergoing delayed THA after failed fixation, the mean Harris Hip Score improved from 53.2 preoperatively to 85.5 at 6 months postoperatively. These findings suggest that THA can provide satisfactory short-term functional outcomes in selected patients with ballistic hip injuries, either as an acute procedure or as delayed reconstruction following failed fixation [16].

Earlier evidence also supports the feasibility of delayed THA after ballistic hip injuries. Pazarci et al. reported a retrospective series of 10 patients who underwent THA following gunshot injury to the hip. The mean interval between injury and arthroplasty was 4.9 months (range, 3–8 months), and the mean Harris Hip Score improved from 25.2 preoperatively to 65.8 at final follow-up. Outcomes were influenced by injury severity and contamination; patients with associated intestinal injury had poorer clinical outcomes and a higher risk of postoperative infection. These findings emphasize that the presence of visceral injury and potential contamination of the hip joint should be considered when determining the timing of arthroplasty [17].

Several case reports have further described staged or delayed THA following severe ballistic hip injuries. Bell et al. [18] reported a patient with comminuted femoral head and neck fractures and retained intra-articular bullet fragments who underwent initial irrigation and debridement, antibiotic cement spacer placement, systemic antibiotic treatment, and subsequent conversion to THA. The staged strategy was selected because of extensive soft-tissue injury and contamination and was intended to optimize local conditions before definitive arthroplasty.

Other reports have similarly described staged reconstruction using initial debridement, temporary stabilization, and antibiotic spacers followed by delayed THA after resolution of the acute soft-tissue injury and exclusion of persistent infection [19, 20].

More recently, case-based literature has continued to support staged reconstruction for complex ballistic hip injuries with extensive acetabular or femoral head destruction. These reports describe damage-control surgery followed by temporary antibiotic spacer placement and delayed definitive THA after optimization of the soft-tissue and bony environment. Although such reports cannot establish comparative efficacy, they illustrate the potential role of staged reconstruction when acute implantation is deemed unsafe due to contamination, extensive soft-tissue injury, or uncertainty about infection control [21].

### Acute versus delayed or staged arthroplasty

The available ballistic-specific literature, therefore, suggests two principal approaches to arthroplasty. Acute or early primary THA may be considered when the femoral head and neck are irreparably damaged, and stable reconstruction is not achievable, provided the patient is physiologically suitable and the local soft-tissue status and contamination status permit implantation. Conversely, delayed THA may be performed after failed fixation, progressive post-traumatic joint destruction, or development of symptomatic post-traumatic arthritis. A staged approach with initial debridement and antibiotic spacer placement may be particularly appropriate when substantial contamination, retained intra-articular material, severe soft-tissue injury, or concern for occult infection makes immediate definitive arthroplasty undesirable [18–21].

Importantly, the available ballistic-specific evidence does not establish the superiority of acute primary THA over delayed or staged reconstruction. The published literature consists predominantly of small retrospective series and individual case reports, with substantial heterogeneity in injury severity, associated visceral and pelvic injuries, contamination, indications for arthroplasty, timing of surgery, and duration of follow-up. Consequently, treatment decisions should be individualized rather than based on a fixed time-based algorithm.

### Principles extrapolated from non-ballistic femoral neck fracture literature

Because ballistic-specific THA evidence remains limited primarily to small case series and retrospective cohorts, many technical recommendations must be extrapolated from the broader literature on failed fixation of conventional blunt-trauma femoral neck fractures. Extrapolating from these data, conversion THA is recognized as a technically demanding salvage procedure due to retained hardware, scarred soft-tissue planes, and femoral cortical defects. These factors often necessitate the use of revision-type or bypass femoral stems to reduce the risk of intraoperative or postoperative periprosthetic fracture [22]. Despite these surgical challenges, conversion to THA significantly improves functional outcomes, with Harris Hip Scores often rising dramatically [23].

Given that patients sustaining ballistic hip injuries are predominantly young adults, THA is generally favored over hemiarthroplasty to maximize long-term functional longevity [24]. In this active demographic, dual-mobility THA components represent an appealing design for both primary and revision cases, as they demonstrate lower dislocation and revision rates compared to conventional THA designs [25–27]. It must be noted, however, that dual-mobility implants may increase the incidence of heterotopic ossification, and long-term survivorship data comparing them to conventional THA in the young trauma population remain undefined [28].

### Outcomes and complications

Ballistic femoral neck fractures are associated with elevated complication and revision rates compared with many other hip fractures, regardless of the initial treatment strategy. Nearly 40% of patients undergoing ORIF experience at least one post-operative complication, including infection, implant-related problems, non-union, and avascular necrosis [1, 2, 4, 5, 8, 11]. When fixation fails, conversion to THA is often required to salvage joint function. While conversion arthroplasty provides reliable pain relief and functional improvement, it carries a higher intraoperative complication profile and a higher risk of infection compared to primary elective THA [16, 22, 23].

Functional outcomes remain inadequately characterized in large cohorts. Still, existing data suggest that poor healing and repeated interventions impair the return to pre-injury activity levels, particularly in young, physically active patients. Persistent pain, limp, and reduced range of motion are commonly reported in those with non-union or avascular necrosis, reinforcing the need for clear prognostic stratification at presentation [2].

## Conclusion

Current evidence supports several practical implications for managing ballistic femoral neck fractures:

Ballistic femoral neck fractures should be approached within the broader context of polytrauma, with systematic evaluation for vascular and additional skeletal injuries.

ORIF remains the preferred option for many displaced fractures, but clinicians should counsel patients about the relatively high risk of non-union and complications (around 40%).

Early consideration of acute or early THA may be appropriate in unstable or comminuted fractures or in patients with significant soft-tissue or vascular compromise. However, this strategy is not yet firmly evidence-based in large cohorts.

Larger multicenter prospective studies are needed to define optimal indications for ORIF versus primary arthroplasty and to refine prognostic criteria (e.g., fracture pattern, vascularity, degree of displacement) specific to ballistic femoral neck fractures. Management should be individualized, with careful weighing of fracture stability, patient age, pre-injury function, associated injuries, and expected compliance with the postoperative program.

## Data Availability

Data sharing does not apply to this article, as no datasets were generated or analyzed during this study.
